# Theory and Guidelines for the Application of the Geophysical Sensor EM38

**DOI:** 10.3390/s19194293

**Published:** 2019-10-03

**Authors:** Kurt Heil, Urs Schmidhalter

**Affiliations:** Chair of Plant Nutrition, Technical University Munich, Emil-Ramann-Str. 2, D-85350 Freising, Germany; schmidhalter@wzw.tum.de

**Keywords:** conductivity modelling, influence of external and internal factors, measurement modes, soil mapping, spatial prediction

## Abstract

Characterization of spatial soil variability is key for a better understanding of soils. To arrive at such information geophysical techniques have been used in the last two decades. Due to its easy handling, the geophysical sensor EM38 has widely been used to characterize agricultural areas. The theoretical background and usage of the EM38 is described, and based on multifaceted applications, the interpretation of the results as well as optimized steps for using it are outlined. Common principles and models of the apparent electrical conductivity (EC_a_) and strengths and limitations of this technique (calibration and temperature effects) are described as well as additional applications, such as the magnetic susceptibility, a comparison of measurements in vertical and horizontal modes, the use of weighted depth information and the influence of measurement conditions are addressed. Further a comparison of EM38 with other proximal soil sensors and fusion with other devices is described. The study reveals that EM38 is useful because the readings can reflect many different soil parameters.

## 1. Introduction

The characterization of the spatial soil variability is key for a better understanding of soils in the landscape. During the last two decades, an increasing number of techniques to detect the heterogeneity of soil properties have been described. Electrical resistivity/conductivity and permittivity based methods are often used in soil science and related research. The high-frequency electromagnetic waves of the permittivity methods are used to determine the velocity and reflection coefficients with GPR (ground penetrating radar). Main target is the soil water content [1,2]. Electrical resistivity/conductivity methods are based on the ability of a material to conduct electrical current. There are two techniques used to measure soil conductivity and resistivity: non-contact system (electromagnetic induction, EMI) and contact electrode measurements [3]. The first method uses a combination of two coils, a transmitter coil with an alternating current and a receiver coil which measures the generated magnetic field. The contact electrode (galvanic coupling) involves devices (at least four electrodes) that direct electrical current into the soil through insulated metal electrodes that penetrate the soil surface. In this case, the devices measure the current between the two receiver electrodes [4]. The primary unit of measurement is electrical resistivity (ER_a_) in Ohm m^−1^. The correct term in geophysics for these methods is “direct current” despite the fact that an alternating current is used. This is the consequence of relatively low frequencies (<1000 Hz) used with ground resistivity methods compared to EMI or capacitive methods (>10000 Hz) [5,6,7].

Since 2001, an increasing number of investigations and applications using the conductivity meter EM38 (Geonics Ltd., Mississauga, Ontario, Canada) have been described. In the nineties, approximately three publications were released per year. This number has increased in the last decade to approximately 10 publications per year. The primary field of application is soil science, followed by agriculture and water content. Additionally, in many countries the EM38 device is commercially used (New Zealand (www.nzcpa.com), Denmark (www.gpsagro.dk), Sweden (www.analycen.se) and Norway (www.planteforsk.no)) [8].

The aim of this study is to outline basic principles and the practical use of EM38. This should improve the reliability, consistency, and compatibility of apparent electrical conductivity (EC_a_) survey measurements and their interpretation. Additionally, the understanding and interpretation of EC_a_ survey measurements is deepened. For each aspect, detailed descriptions and guidelines are given. The objectives of this study are to outline:

- Principles of conductivity measurements;
- Important general models relating EM38-EC_a_ to its contributing factors;
- Practical application of EM38 (calibration, influence of temperature, measurements using different modes, use of depth-weighted or non-weighted soil properties);
- Comparisons with other conductivity/resistivity sensors;
- Fusions with other sensors.

## 2. Theory

### 2.1. Principles of Conductivity Measurements

The distribution of particles and pores determines the amount of conductivity. However, the conductivity is also affected by the concentration of electrolytes in the pore water [9,10,11,12]. Soil conductivity depends on the presence of dissolved inorganic solutes in the aqueous phase consisting of soluble and readily dissolvable salts in soil, including charged species (e.g., Na^+^, K^+^, Mg^2+^, Ca^2+^, Cl^-^, HCO^−3^, NO^−3^, SO_4_^2-^ and CO_3_^2-^), non-ionic solutes, and ions, which combine to form ion pairs [6]. In addition to soil texture, soil temperature, bulk density, water content and soil organic matter (e.g., [9,10,13,14,15] are influential. Ions inside the electrical diffuse double layer of clay particles are capable of conducting electricity, even with low water contents [9,16]. Therefore, clay minerals significantly contribute to soil conductivity [13]. Some soil particles do not conduct electricity and act as insulators reducing the current flow, e.g., quartz, mica, calcium carbonate, and gypsum [17].

**Figure 1:**
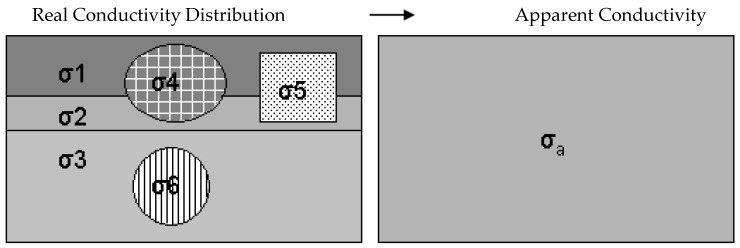
The concept of true and apparent conductivity [4]. σ_1_-σ_3_: The conductivity composed of several soil horizons/layers, σ_4_-σ_6_: liquid, solid-liquid, solid soil phases, and σ_a_: apparent conductivity.

Soil is not a homogenous three-dimensional room, soil consists of an irregular distribution of pores (filled with water/air) and solid substances. Therefore, in geophysics, the term apparent conductivity is used. If the soil is perfectly homogeneous and deep (regular distributed pores and substance, no heterogeneity with increasing depth), the apparent conductivity would be the true conductivity (Figure 1). Three pathways of current flow contribute to EC_a_: 1. liquid, 2. solid-liquid and 3. solid (Figure 1). 

### 2.2. Models of EC_a_

In this section we describe the frequently applied EC_a_ models. Several of these models have been constructed for saturated conditions [18,19,20]. One of the earliest physical models was developed by Maxwell (1881) which is generally written in an asymmetric form (Equation (1)). If the solid particles show no conductivity, the relationship is reduced to Equation (2) (after [21]. This formula determines the conductivity of a heterogeneous medium consisting of spherical particles and a suspension [22,23]

(1) $$\frac{EC_{a}-EC_{w}}{EC_{a}+2EC_{w}}\;=\;\int \frac{EC_{s}-EC_{w}}{EC_{s}+2EC_{w}}$$

(2) $$\;\frac{EC_{a}}{EC_{w}}\;=\;\;\;\frac{2\eta }{3-\eta }$$

EC_a_—apparent electrical conductivity of the bulk soil [dS m^−1^]
EC_W_—specific electrical conductivity of the soil water [dS m^−1^]
EC_S_—electrical conductivity of solid soil [dS m^−1^]
η—porosity

As described by Friedman (2005) [21], this simple mathematical model over-predicts the measured conductivities of mono-sized glass beads. 

The simplest relationship is given by Archie for brine-saturated rocks (1942):

(3) $$EC_{a}=\frac{1}{F}\times EC_{w}\;\;\;\;\;\;\frac{EC_{a}}{EC_{w}}=\;\eta^{m}\;\;\;\;\;\;\;\mathrm{Archie}’s\;\mathrm{Law}$$

m—material dependent empirical exponent, cementation index
F—formation factor

According to Shah and Singh (2005) [23], m depends on the particle size characteristics of the soil. EC_a_ is linearly dependent on ECw. The slope is determined by the factor F. Archie (1942) [18] found characteristic m values of 1 to 3 for unconsolidated sands and 0 to 2 for consolidated sands. The porosity ranges from 0 to 1 for clean sand and 0 to 6 for tuff particles [21]. 

For unsaturated porous media, Archie’s law is expressed as:

(4) $$EC_{a}\;\;=\;c\times EC_{W}\;\times \;\eta^{m}\times S^{A}$$

and is written in a generalized form (assuming m=A and a non-significant influence of η):

(5) $$\frac{EC_{a}}{EC_{W}}=\;c\;\times \Theta^{A}\;\;=\frac{1}{F}$$

C, A—parameters
S—degree of saturation
Θ—volumetric moisture content

Additionally, the effect of particle conductivity EC_P_ on EC_a_, which is important for dry soils (i.e., θ = 0), can be incorporated in the generalized Archie’s law by adding EC_P_ to Equation (6). The authors noted that for most soils, EC_P_ is so small that it can be ignored. 

The derivations of EC_a_ are based on the assumption that there is negligible contribution of surface conductance (adsorbed ions, EC_S_) to EC_a_. The consideration of structural aspects is only valid in coarse textured soils or in soils where EC_W_ is sufficiently high to dominate EC_a_ [21]. The author described that, at low salinity in dominantly fine textured soils (EC_W_ <4 dS m^−1^), the EC_a_-EC_W_ relationship is nonlinear. 

Based on the formula from Archie (1942) [18], Sen et al. (1988) [20] introduced the influence of surface charge (Q_v_).

(6) $$EC_{a}=\frac{1}{F}\times \left(EC_{w}+EC_{w}\times \frac{a\times Qv}{EC_{w}+b\times Qv}\right)+c\times Qv$$

Q_v_—charge per unit pore volume (surface charge density) [mol L^−1^]
a, b, c—constants [S m^−1^ mol^−1^]

The constants a, b and c depend on the mobility of the ions close to the surface of the solid phase. 

Sen et al. (1988) [20] used the following simplifications:

- the term (c* × Q_V_) conductivity of the solid soil is similar to Rhoades [24] (1989) and is negligible,
- the parameter a is estimated by a = 1,93 × m_Sen_, where

(7) $$m_{sen}=1.67+1.953\sqrt{\frac{CEC}{100}}$$

m_Sen_—m after Sen
CEC—cation exchange capacity [mmol kg^−1^]

and

- (b* × Q_V_) is replaced by 0.7 S m^−1^.

These parameters lead to the following formula:

(8) $$EC_{a}=\frac{1}{F}\times \left(EC_{w}+EC_{w}\times \frac{1.93\times m_{Sen\;\times }Qv}{EC_{w}+0.7\times Sm^{-1}}\right)+c\times Qv$$

As described by Friedman (2005) [21], this empirical expression is reasonably successful in describing the EC_a_ of clay-bearing sandstones. 

Mualem and Friedman (1991) [25] presented a model for saturated and unsaturated conditions (Equation (9) as cited by [23].

(9) $$EC_{a}=EC_{w}\times \left(\frac{\Phi^{\eta +2}}{\Phi_{sat}}\right)$$

Φ = (Θ-Θ_b_) reduced volumetric moisture content
Φ_sat_ = (Θ_sat_ -Θ_b_) reduced saturated volumetric moisture content
Θ_b_ bound volumetric moisture content [cm^3^ cm^−3^]

Günzel (1994) [26] modified the model of [20] to estimate EC_a_ from different soil parameters under unsaturated conditions given as follows:

(10) $$EC_{a}=\;\;\theta^{m}\;\times \left(EC_{w}+EC_{w}\times \frac{0.193\times m\times BD\times CEC}{\;\left(EC_{w}\;\;+0.7\right)\times \theta \;\;\;\;\;\;\;\;}\right)$$

BD—bulk density [kg l^−1^]

In addition to Archie, Rhoades presented models which have been frequently applied, such as the simplified approach of [10]:

(11) $$EC_{a}=\;EC_{w}\times \theta \times t+EC_{s}$$

t—transmission coefficient: t = a × Θ + b

Rhoades et al. (1976, 1989) [20,24] formulated an electrical conductance model as a function of the mobile and immobile water content, bulk density, and conductivity of soil water and soil surface to calculate salinity.

(12) $$EC_{a}=\left(\frac{\left(\theta_{ss}+\theta_{ws}\right)2\times EC_{ws}\times EC_{s}\;\;\;\;\;\;}{\left(\theta_{ss}+EC_{ws}\right)+\left(\theta_{ws}+EC_{s}\right)}\right)+\left(\theta_{sc}\times EC_{s}\right)+\left(\theta_{wc}\times EC_{wc}\right)$$

Θ_WS_—volumetric soil water content in the soil-water pathway in fine pores (immobile water, series coupled) [cm^3^ cm^−3^]
Θ_WC_—volumetric soil water content in the continuous-liquid pathway in medium and coarse pores (mobile water) [cm^3^ cm^−3^]
Θ_SS_—volumetric content of the surface-conductance solid phase [cm^3^ cm^−3^]
Θ_SC_—volumetric content of the solid phase [cm^3^ cm^−3^]
EC_WS_—specific electrical conductivity of the soil water pathway in fine pores (series coupled) [dS m^−1^]
EC_WC_—specific electrical conductivity of the continuous-liquid pathway in medium and coarse pores [dS m^−1^]

This equation shows that EC_a_ is a function of soil physical and chemical properties. The different pathways connect electrical resistances. Assuming that the solid pathway is negligible because EC_S_ << EC_WC_ and that the conductivity is not influenced by pore size, the equation becomes simpler:

(13) $$EC_{a}=\;\left(\frac{\left(\theta_{s}+\theta_{ws}\right)2}{\theta_{s}}\times EC_{s}\right)+\;\left(\theta_{w}-\theta_{ws}\right)\times EC_{wc}$$

Θ_W_—Θ_WS_ + Θ_WC_ = total volumetric water content [cm^3^ cm^−3^]
Θ_S_—saturated volumetric water content

Rhoades et al. (1989) [24] investigated soils with a strong relationship between EC_S_ and soil texture. Θ_S_ is closely correlated with bulk density, and EC_a_ is a function of the bulk density, Θ_W_ and EC_W_. The authors recommended performing EC_a_ measurements at higher water contents (i.e., field capacity) to assess the amount of EC_w_. 

The formulas from [18,19,20,24] are useful to detect the salinity. McBratney et al. (2005) [27] termed such soils hyper-electrolytic because there is a large amount of electrolytes in relation to the charge of the soil. If there is a smaller amount of electrolytes compared to the charge (ortho-electrolytic, [27], the formulas can be used to map other soil properties. The authors presented a theoretical calibration model for EC_a_ assuming ortho-electric situations and that the conductivity of the underlying material is much smaller than that of the upper soil:

(14) $$EC_{a}=\;A\times {\left(\frac{\mathrm{clay}}{100}\right)}^{a}\times B\times {\left(\frac{\Theta }{\Theta s}\right)}^{b}\times C\times {\left(\frac{\mathrm{CEC}}{{\mathrm{CEC}}_{0}}\right)}^{c}\times D\times {\left(\frac{\rho b}{\rho 0}\right)}^{d}\times {\left(1+\exp\left[T-\frac{293}{293}\right]\right)}^{e}$$

clay—percentage of clay [g kg^−1^]
CEC_0_—cation exchange capacity of the reference soil material
ρ_b_—bulk density
ρ_0_—bulk density of the reference soil material
T—absolute soil temperature
A, B, C, D—empirical constants relating the contribution of each component to EC_a_
a, b, c, d, e—empirical constants accounting for the nonlinear relationships between soil properties and the EC_a_

If temperature correction is assumed and ρ_b_ is reflected in Θ, the equation becomes:

(15) $$EC_{a}=\;\kappa \times \left(\frac{clay}{100}\right)\times \left(\frac{\Theta }{\Theta s}\right)\times \left(\frac{CEC}{CEC_{0}}\right)$$

κ—scaling factor [mS m^−1^] which summarizes the A, B, C, D, E constants

Heil (unpublished) calculated site-specific empirical models where the target variables were the EM38 v- and h-readings. However, other investigations [28] under controlled conditions with soil samples in cylinders from the same study area, resulted in more complex relationships:

(16) $$\begin{array}{l} \sqrt{{\mathrm{EC}}_{\mathrm{av}}}=3.91+0.000042\times {\mathrm{conductivity}\;\mathrm{pore}\;\mathrm{water}}^{2}\;+14.81\times {\mathrm{clay}}^{2}\\ \;\;+1.73{\;\mathrm{silt}}^{3}\;–0.01\times \;\mathrm{water}\;\mathrm{content}\;–0.45\times \;\mathrm{cultivation} \end{array}$$

(17) $$\begin{array}{l} \;\log{\mathrm{EC}}_{\mathrm{ah}}=0.66+0.003\times \mathrm{conductivity}\;\mathrm{pore}\;\mathrm{water}+0.99\times \mathrm{clay}+0.3\times {\mathrm{silt}}^{3}\\ \;\;+0.006\times \mathrm{water}\;\mathrm{content}\;–0.05\times \mathrm{cultivation} \end{array}$$

where

(EC_av_)—apparent electrical conductivity of bulk soil [dS m^−1^], measured in the vertical mode
(EC_ah_)—apparent electrical conductivity of bulk soil [dS m^−1^], measured in the horizontal mode

The EC_W_, clay, silt, water content and cultivation (integrated farming = 1, organic farming = 0) in both modes were the most important factors of influence. Clay and silt reflect the soil conditions, and the conductivity of pore water and the cultivation reflects the field specific fertilization and the water content. The major factors affecting EC_a_ are the same but their relative importance differs. 

This brief summary of more generally applied models and regionally developed formulas demonstrates that the liquid phase and the porous solid phase are present in all of the models. The form in which these parameters are taken into account differs in a distinct way. Further some variables can be substituted by related variables. One example of these “substitutes” is EC_W_ – EC_S_ – CEC – θ. According to different authors [20,29,30], the contribution of the surface conductivity to EC_a_ increases with an increasing double layer. The concentration of the double layer is mainly determined by several soil parameters, such as water content, pore-fluid chemistry, pH, etc. Nadler and Frenkel (1980) [31] reported that for low pore-solution salinity (i.e., EC_W_ < 0.4 S/m), EC_S_ is not constant and depends on EC_W_. Shah and Singh (2005) [23] stated that the effect of EC_S_ is automatically included in m. These theoretical considerations show that soils with increasing fine granular material are more complex than soils with low EC_a_ readings. 

Durlesser (1999) [13] compared the formula of [26] with his own measurements from the Scheyern site. The influence of BD on the EC_a_ was ten times weaker than CEC, EC_W_ and Θ. CEC, EC_W_ and Θ had approximately the same degree of influence. When the texture was unknown, it was possible to calculate Θ. According to [23], the formula from [26] can be used for soils with lower values of Θ. Shah and Singh (2005) [23] favored the generalized Archie’s law, which can be used for saturated and unsaturated soils. For the investigated soils, the generalized Archie’s law allowed for a better simulation than was obtained by [20,24,26]. 

A newly introduced variable was used for cultivation [32]. It indicates the level of EC_W_ and short-term effective changes of EC_W_ which correspond to a limited degree to other conductance effective soil properties. 

Some authors [23,25,31] discussed additional variables, such as the ionic mobility that depend on the distance to the particle surface. The mobility and the contribution to the conductivity are lower in the double layer as in the center of the pores. According to [23], the formula discussed in [25] accounts for this observation. 

Friedman (2005) [21] introduced the role of various attributes of soil and its solution to determine the EC_a_. This fact concerns EC_a_-EC_w_ and, therefore, saline soils. Non-spherical particle shapes and broad particle-size distributions decrease the EC_a_. When the non-spherical particles have some preferential alignment in space, the soil is anisotropic, meaning the EC_a_ depends on the direction in which it is measured. The influence of temperature on the EC_a_ is stronger than in free solution. These findings limit the applications of EC_a_-EC_w_ models. To solve these problems, physical models are used, which describe the contribution of dissolved ions, mineral surface properties and solution chemistry. When these models are not available, therefore, site-specific EC_a_ (EC_w_, θ) calibrations are necessary to determine accurate relationships to EC_w_. 

Kühn et al. (2008) [33] explained the variation in EC_a_ to a large degree by the below-mentioned factors with an R^2^ of up to 61%. The soil parent material in their investigation area consisted of glacial till, fluvial deposits (mainly sands), loess and clay stones and Holocene and Weichselian alluvial sediments. The soil organic matter and CaCO_3_, secondarily clay and gleyic horizons were significantly related to the EC_a_. The authors could combine the geological map unit and the EC_a_, which made it possible to determine the most important soil properties affecting the EC_a_ by interpreting the geological maps.

### 2.3. Principles of EM38 Measurements 

The calculated values of the EC_a_ are a function of calibration, coil orientation, and coil separation. The separation between the coils determines the volume of material detected by the instrument. This concept is often extended to include a depth of penetration of the technique, with larger separations producing greater depths of investigation [34].

**Figure 2:**
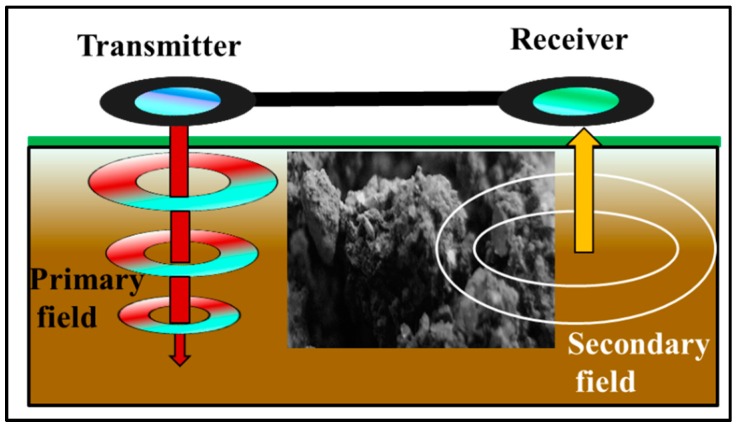
Schematic construction of the EM38 measurement principle (transmitter, receiver, primary and secondary field).

The ground conductivity meter EM38 is composed of a transmitter and a receiver coil installed 1.0 m apart in a non-conductive bar in the opposite ends of the instrument. It operates at a frequency of 14.6 kHz. The transmitter coil is energized with an alternating current from a 9 V battery, which generates a time-varying magnetic field in the earth. This magnetic field causes the current to flow in the soil, producing a secondary magnetic field (Figure 2). The measurement is expressed by the ratio of the primary field (H_p_) to the secondary field (H_s_) (Equation (18)). It is a function of the different conductivities in the subsoil and other factors, such as the orientation and distance of the coils, operating frequency and the magnetic susceptibility [9]. 

Ground conductivity meters operate within the limits provided by a low induction number [35].

(18) $$EC_{a}=\frac{4}{2\Pi \mu_{0}\;\;f^{2}\;\;s\;}\;\;\times \;\left(\frac{H_{s}}{H_{p}}\right)$$

H_p_—primary magnetic field (A m^−1^)
H_s_—secondary magnetic field (A m^−1^)
f—frequency of the current (Hz),
μ_0_—magnetic permeability of air (4π10^−7^ H m^−1^);
s—intercoil spacing (m).
Π—2 πf

A detailed discussion of the equipment and its operation can be found in [36].

**Figure 3:**
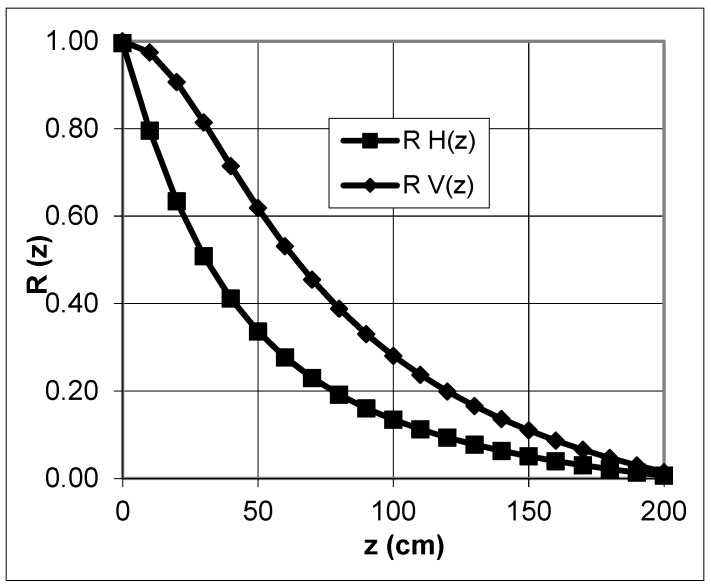
Calculated, relative, cumulative contribution versus depth for vertical (RV(z)) and horizontal (RH(z)) orientated dipoles.

**Figure 4:**
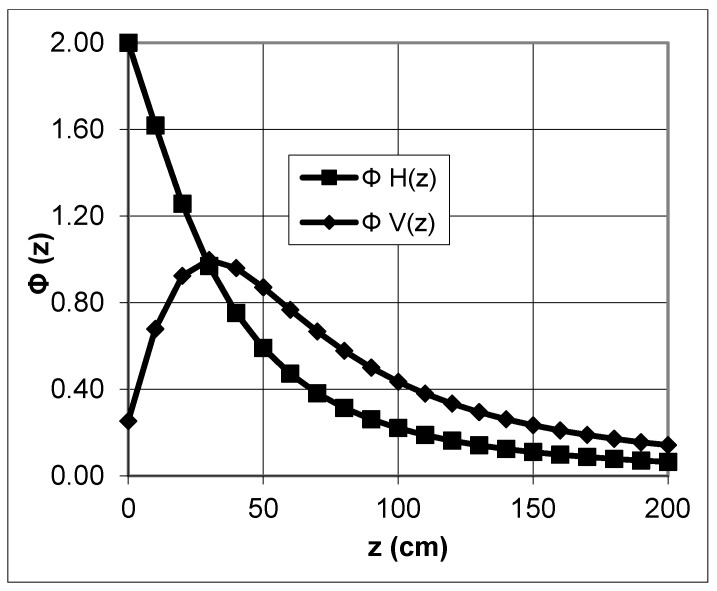
Comparison of calculated relative responses for vertical (ΦV(z)) and horizontal(ΦH(z)) oriented dipoles.

The investigated depth range depends on the coil separation within the instrument. The coil distance is fixed using the EM38. Only the orientation of the coils can be changed. This depth-weighted nonlinearity is shown in Figure 3 and Figure 4. The figures illustrate the cumulative relative contributions of all of the soil electrical conductivities, *R*(*z*), for a homogeneous conductive material below a normalized depth of *z* based on Equations (19) and (20) from [9] for both dipoles, respectively.

(19) $$R_{v}\left(z\right)=\;\frac{1}{{\left(4z^{2}+1\right)}^{0.5}}$$

(20) $$\;R_{H}\left(z\right)=\;{\left(4z^{2}+1\right)}^{0.5}-\;2z\;$$

z—depth [m]
R—cumulative relative EC_a_

The terminology “vertical” (EC_av_) and “horizontal” (EC_av_) are used differently.

- Vertical coils and vertical magnetic dipoles are called HCP (horizontal coplanar).
- Horizontal coils (the magnetic dipoles are horizontal) are called VCP (vertical coplanar).

For VCP, the tool lies parallel to the soil surface and for HCP, the instrument is perpendicular. According to [37] Geonics Ltd. (2002) and [9], the orientation of the dipoles has to be eponymous for different modes (vertical or horizontal dipole). Other authors (e.g., [38] use the position of the coils (vertical or horizontal coils). We use the direction described by Geonics Ltd. The sensitivity in the vertical mode is the highest at approximately 40 cm below the instrument and in the horizontal mode reaches a maximum directly below the instrument. 

The absolute determination of the investigation depth is problematic. The measurements can be acquired at an unlimited depth but, in reality, it depends on the electrical contrast. [38] discussed a simple definition. According to the authors, the investigation depth is limited to where the effect of a layer is considered “noise” for the instrument. A threshold of 10% is generally used by the authors. For EM38, the most common definition and simplification is a depth range of up to 1.5 m using the vertical dipole mode. With the horizontal dipole mode, the penetration depth is reduced to 0.75 m [39,40].

## 3. Application of EM38

### 3.1. Performance of EM38 Measurements in Practice

The EM38 measurements can be made quickly and it is relatively easy to obtain many readings with a good spatial resolution. The EM38 is sensitive to proximal metal objects. Therefore, it is recommendable to remove all metallic objects during data collection. A metallic presence will affect the zero setting and also the values during the measurements. Additionally, electromagnetic noise, such as power lines manifests itself in oscillating readings. 

**Figure 5:**
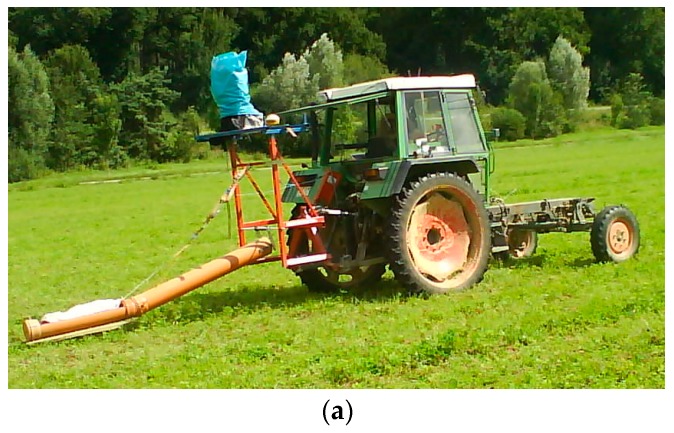

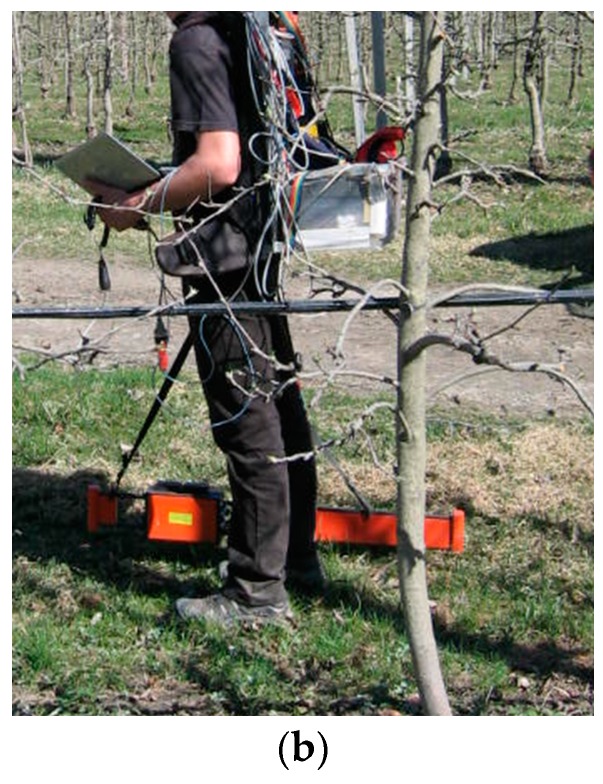
(**a**) EM38 mounted on a metal-free sledge pulled by a tractor (constructed after [44] Corwin. and Lesch 2005c); (**b**) hand guided measurement.

For wide area measurements, the sensor is mounted on a metal-free sledge and pulled behind an all-terrain vehicle equipped with a GPS receiver and data collection computer. In the literature, several forms of vehicles are described and pictured (e.g., [41,42,43]. Another possibility is a hand guided measurements (Figure 5). With a GPS on the back, this method is particularly suitable for smaller area, steeper fields or areas that are not to be travelled.

To assure the reliability, consistency, and compatibility of EC_a_ data, [42,43] developed a survey protocol, which accounts for all of the relevant information regarding measurements and interpretation. For surveys, in line with the joint project iSoil (http://esdac.jrc.ec.europa.eu/projects/guidelines, 2017) we recommend the following steps:

(i) intended objective;
(ii) evaluation (stochastic and/or deterministic analysis, spatial statistical analysis);
(iii) site description (relief, common available soil information);
(iv) current land use (kind and amount of fertilization);
(v) specification of geo-referenced EC_a_ survey design (point-wise or distance of tracks, measurements of specific zones within areas, e.g., field corners);
(vi) soil sample design (number of samples, sampling depths, sampling method, depth of grid sampling or orientation after EC_a_ readings, soil maps, topography); and
(vii) crop harvesting design.

#### 3.1.1. Calibration of EM38

Although this tool is very popular, some weaknesses exist [5,45]. One of the greatest disadvantages during the measurements is the drift of the Q/P (Quad phase) and I/P (Initial phase) readings. The I/P value measures the sensitivity of the receiver electronics to the primary signal induced by the transmitter [37]. For optimum accuracy, the I/P reading should be maintained at zero using the EM38 controls [37]. Lück et al. (2000) [11] described the incorrect setting of the I/P switch, which changes the v- and h-readings and their relationship. The Q/P adjustment shows the EC_a_ readings. With the Q/P, the instrument is set to zero on the ground, and in a special v-h-constellation, the instrument is positioned at approximately 1.5 m [37]. The drift is often explained by changes in the ambient temperature and the sensor’s temperature [41,46]. Robinson et al. (2004) [47] demonstrated that differential heating of EM38 is one cause of drift and erroneous readings. 

The user’s manual [37] suggests nulling at the beginning of each day and then three or four times per day. When used as a mobile unit linked with a GPS receiver, a re-nulling is frequently too complicated. One way around this complication may be re-nulling the I/P reading at fairly frequent intervals, such as every 15–30 min. Sudduth et al. (2001) [41] suggested applying a manufactured version of self-corrections of I/P drifts using temperature changes. 

In addition to the air temperature effect, the soil temperature has a negative influence on EC_a_ values collected over the course of a single day because large amplitudes are present in the upper soil areas. The results from [46] indicated that such assumptions are valid under the conditions during their study (fluctuations less than 10°C during the day at a depth of 5 cm) and when frequently nulling the I/P readings. According to [37], the drift of the temperature of the Q/P mode results in a change of 0.15 mS m^−1^ per °C, and the in-phase-component denotes 2.5 mS/m per °C [11]. 

Sudduth et al. (2001) [41] reported a drift of approximately 3 mS m^−1^ per h. This could represent over 10% of the total EC_a_ variation in some fields. To reduce this, the authors recommended (i) re-zeroing the instrument I/P or (ii) using a calibration transect to monitor drifts over the course of a survey and adjust EC_a_ readings for the drift. To ensure quality data, re-zeroing or collecting data on a calibration transect should be conducted approximately every half-hour or at least once every hour.

Based on the procedures suggested by [41,48] developed equations to correct the drift of EC_a_ and magnetic susceptibility depending on the environmental temperature.

Optimized procedures include the following steps: (1) On a fixed point, the measurements are performed for approximately 20 min. (2) The first minute of the measurement is chosen as a reference value, representing readings without the influence of temperature. (3) The difference between the ongoing readings and reference EC_a_ from the start of the measurement period provides residues. (4) This residues are related to the measurement time and equations are calculated. (5) With these equations, all the measurements are then corrected. 

The authors found out that quadratic relationships deliver the best results to detect masked anomalies caused by buried metallic pipes and electrical cables. 

Re-nulling during the course of a survey produces jumps in the readings and correcting the recorded temperature variations may be too time-consuming. In our experience, an appropriate way to reduce such an influence includes the following steps: (i) Allowing the system to be in equilibrium with the ambient at the beginning of the measurement and (ii) avoid direct sunlight during the survey, particularly at higher temperatures. Changes in direct sunlight and shadows can produce an enormous drift. Therefore, it is strictly recommended to perform the survey with EM38 in a closed box, preferably made out of wood. (iii) It is often necessary to correct for any indirect effects. The suggested procedure is the point wise EC_a_ determination at the beginning and end of the measurements on the same site. The calculated drift is added or subtracted. Such information is not given in the user’s manual.

#### 3.1.2. Consideration of Soil Temperature

Electrolytic conductivity increases at a rate of approximately 1.9% per 1 °C increase in temperature. Due to this, all EC_a_ values are recalculated to a temperature of 25°C (EC_25_). Different formulas were developed. The function described by [49] and modified by [42] is often used.

(21) $$EC_{25}\;=\;EC_{a}\times \left(0.447+1.4034\times {\exp\;}^{\left(-\frac{T}{26.815}\right)\;\;\;\;\;\;}\right)$$

Tsoil temperature [°C]
EC_25_—EC_a_ recalculated to a soil temperature of 25°C

A ratio model is commonly used to correct EC_a_ measurement to a standard temperature (e.g., [50,51].

(22) $$EC_{25}=\frac{EC_{T}}{1+\vartheta \left(T-25\right)}\;\;\;\;\;\;\;\;\;\;\;\;$$

EC_T_—EC_a_ [mS m^−1^] measured at the actual soil temperature T [°C]

where EC_T_ is the soil electrical conductivity at the actual temperature T, and ϑ is the temperature compensation for the slope. The commonly used value for ϑ is 0.0191C^−1^. 

Durlesser (1999) [13] developed the following formula for the special climatic and soil conditions at Scheyern [28]:

(23) $$EC_{25}\;=\;EC_{a}\times \left(0.447+1.69\times {\exp\;}^{\left(-\frac{T}{2621.0}\right)}\right)$$

Rhoades et al. [52] (1999) presented another formula:

(24) $$f_{t}\;=\;1\;-\;0.20346\;\left(T\right)+\;0.03822\;\left(T^{2}\right)-\;0.00555\;\left(T^{3}\right)$$

where

T—[temperature in °C-25]/10
f_t_—formation factor

Conductivity at 25 °C is calculated as follows:

(25) $$EC_{25}=f_{t}\times EC_{t}$$

where EC_t_ is the soil electrical conductivity at the measured temperature T.

McBratney et al. (2005) [27] were the only authors who integrated a temperature correction in their EC_a_ equation (Equation (14)). 

Ma et al. (2011) [53] compared different formulas with data from the US Salinity Laboratory. The results were surprising. The authors found several wrong equation citations. Following these comparisons, the following conclusions are reached:

- The exponential model of [49], as modified by [42], shows best results (Equation (21)). The authors suggested that practitioners should use this equation to correct EC_a_ readings ranging from 3 to 50 °C to provide measurements referenced to 25 °C (EC25);
- The ratio model (Equation (22)) is also applicable between 3 °C and 47 °C.
- The model of [53] is only applicable for a 15 to 35 C° range, for which it was originally designed.

Slavich and Petterson (1990) [17] considered seasonal changes in soil temperature. When using the EM38 over an extended period of time in the field, the seasonal variation in soil temperature is likely to affect EC_av_ and EC_ah_ values. The authors calculated temperature correction factors for various times of the year for NSW (Australia). They found that, although the correction factors varied widely with the season, they were not significantly affected by the shape of the EC_a_ profile. A correction procedure for seasonal variation in soil temperature was outlined by [54]. Only a small amount of information regarding the annual variation in soil temperatures was necessary to develop an effective correction factor for EC_a_ readings. Such data exists for many of the major agricultural regions in Australia. 

Recently Geonics Ltd. developed a new coil technology, with a temperature compensation circuitry to reduce the temperature-related drift characteristics as compared with the preceding generation of EM38 instruments.

#### 3.1.3. Magnetic Susceptibility

The EM38 allows additional measurements of the apparent magnetic susceptibility (in-phase component). When a magnetic field is present [55,56], metal materials become magnetized. The measurement is performed as per unit volume (K) or as a mass normalized susceptibility (χ) [55]. When measured with the EM38, the values are given as the ratio of the primary to the secondary field in parts per thousand (ppt) [57]. Therefore, the in-phase component is considered a measure of magnetic susceptibility [58]. However, it is generally a rarely used variable. 

The magnetic susceptibility is influenced by the following factors [55]:

- some oxides and hydroxides;
- topsoil is much more magnetic than subsoil layers;
- human activities enhance topsoil properties (organic matter, porosity, soil temperature as a consequence of more organic matter), meaning cultivated soils are often more magnetic than non-cultivated areas; and
- burned material enhances magnetic susceptibility.

The magnetic susceptibility of near-surface materials does not normally vary significantly over short distances (e.g., a few meters) [59]. Soils sometimes have higher values than the parent rocks because of weathering processes. Detecting 250 years of old bricks with magnetic susceptibility was described by [60]. These data can help to locate buried soil horizons, cultural strata, and answer questions regarding cultural formation and post-depositional processes [55,61].

Wynn (1990) [62] used the magnetic susceptibility of soils in the 100 to 10,000 Hz range to explore archaeological areas. This application was useful because it can provide information on the presence of disturbed clay- and sulfide-rich (particularly pyrite) horizons in areas with human influence. 

The EM38 was able to detect the remains of a 17th century castle in the pasture fields of Vinkem. The wall foundations were clearly visible because of a strong anomaly in the in-phase response of the 1 m vertical coplanar orientation, which was caused by the enhanced magnetic susceptibility of the bricks. In the horizontal coplanar orientation, the anomalies were less clear and showed both positive and negative responses, which were likely caused by the change in sign of the response at a certain depth. A remarkable fact was that the quadrature-phase response did not show the walls in horizontal coplanar orientation.

Ernenwein and Hargrave (2009) [57] listed the advantages and disadvantages:

- The EM38 is prone to drift and the data are sometimes difficult and time consuming to process;
- The magnetic susceptibility is not limited to the direction and strength of the earth’s magnetic field and can detect features regardless of geometry;
- These data are absolute values, rather than a collection of positive and negative poles.

EM38 and the DUALEM-21S were used by [56]. The magnetic structures of buried objects resulted in clear measured anomalies with a high resolution. Magnetic susceptibility and EC_a_ readings were used by [48] to detect electrical cables and metallic pipes. The development of site specific equations to correlate the environmental temperature enhances the quality of registrations of these soil anomalies. 

### 3.2. Comparison of EC_av_ with EC_ah_

Comparisons of both modes show close relationships with high coefficients of determination values [63,64]. 

Nogues et al. (2006) [65] gave an example from saline and non-saline Spanish soils with the following relationships:

(26) $$EC_{av}=0.54+1.1\times EC_{ah}\;\;\;\;R^{2}=\;0.98,$$

and

(27) $$EM_{av}=0.08+1.05\times EC_{ah}\;\;\;\;R^{2}=\;0.94.$$

Generally, the v-mode values are related to different properties within the whole profile. In case of topsoil calculation, using EC_ah_ is often more realistic. The reason for this phenomenon is the different sensitivity functions of the EM38 [9], which show that the relative response to the signal from the topsoil is larger for EC_ah_ than for EC_av_. 

EC_ah_ delivered better results when estimating the soil depth to a cemented (petrocalcic) horizon [66]. The same result was described by [67] when determining the thickness of A-horizons. Heath et al. (1999) [68] reported that, in Australia, frequent measurements in the h-mode are used. This is caused by the fact that shallow rooting crops (pastures) are objects of investigations. Dalgaard et al. (2001) [69] found similar R^2^ values for the derivation of the clay-content, although the spans of the EC_a_ and clay texture were narrower in the h-mode. Most research does not describe a favorite mode (e.g., [70]. 

Some authors prefer the combination of EC_av_ and EC_ah_ as an average EC_a_-value for the top 100 cm of soil profiles. Norman (1990) [71] and Slavich (2001) [72] suggested that changes in the concentration of salts deeper in the soil profile may significantly affect the average regression equations. Therefore, Slavich (2001) [72] developed regressions for leached and inverted concentrations of salt. 

For an inverted profile where (EC_ah_ > EC_av_)

(28) $$EC_{a}\left(0-60\;\mathrm{cm}\right)=\;1.87\times \;EC_{ah}\;-\;1.87\times \;EC_{av}$$

For a leached profile where (EC_ah_ < EC_av_)

(29) $$EC_{a}\left(0-60\;\mathrm{cm}\right)=\;1.24\times \;EC_{ah}\;-\;0.05\times \;EC_{av}$$

Slavich (2001) [72] argued that EC_av_ and EC_ah_ are generally strongly correlated, and a more valid approach needs to be used.

(30) $$EC_{\left(a-average\right)}=\;\left(\frac{\left(EC_{av}+EC_{ah}\right)}{2}\right)$$

(31) $$\;EC_{\left(h-v\right)}=\;\left(EC_{ah}-EC_{av}\right)\;$$

These measurements are likely to be more weakly correlated and can be interpreted as measures of an average profile value (e.g., salinity, Equation (30)) and profile trend (Equation (31)).

A calculation with a similar intention was presented by [73]. A profile ratio (PR) was created by combining the EC_a_ values measured in the two orientations.

(32) $$\mathrm{PR}\;=\frac{EC_{ah}}{EC_{av}}$$

PR close to 1 indicates a uniform profile, a PR < 1 indicates a more conductive subsoil compared to the topsoil and PR > 1 indicates a decreasing conductivity with depth.

To predict the organic carbon, Martinez et al. (2009) [74] applied a normalized EC_a_ difference (ΔEC_a_), calculated as the difference between the normalized vertical and horizontal dipole EC_a_ values:

(33) $${\Delta \mathrm{EC}}_{a}=\;\left({\mathrm{EC}}_{\mathrm{av}}/\bar{{\mathrm{EC}}_{\mathrm{av}}}\right)\;-\;\left({\mathrm{EC}}_{\mathrm{ah}}/\bar{{\mathrm{EC}}_{\mathrm{ah}}}\right)$$

Vanderlinden et al. (2010) [75] integrated EC_ah_ and EC_av_ maps of different measurement campaigns to derive the management zones. Interpolated data were transformed to relative differences ($\delta_{ij}$).

(34) $$\delta_{ij}=\;\frac{{\mathrm{EC}}_{\mathrm{aij}}-\;{\left({\mathrm{EC}}_{a}\right)}_{j}}{{\left({\mathrm{EC}}_{a}\right)}_{j}}$$

where EC_aij_ is the EC_a_ representing point (pixel) i and survey time j, and EC_aij_ is the spatial average of the field at survey time j. For each location I, the mean relative difference $\delta_{i}$ of the 13 measurement campaigns and its standard deviation ($\sigma_{\;\delta i}$) were calculated to evaluate the temporal persistence or to rank the stability of the EC_a_ patterns. 

### 3.3. Comparison of EC_a_ and Depth Weighted or Non-Depth Weighted Soil Properties

Soil properties should be introduced in calculations with EC_a_. [76] compared three forms of target variables: (i) Depth weighting according to EM38 depth sounding (EC_av_), (ii) depth-weighted average according the thickness of the horizon, and (iii) value from top layer of the profile. EC_a_ correlations with sensor-weighted silt content and cation exchange capacities were generally the highest and most persistent. The coefficients of clay content were different, but the improvement of horizon-weighted values was enhanced to a minor extent. Organic C sensor-weighted relationships were also better. These higher correlations with sensor-weighted data supported the thesis that the weighting corresponds with the linearity of the EM38 depth function. In all listed cases, the relationships to the topsoil values were weaker. Additional results were described by [64]. Depth weighting with the signal distribution of the sensor did not enhance and slightly improved the relationships.

Schmidhalter et al. (2001) [64] compared EC_a_ readings for the v- and h-mode with depth measurements of soil texture and water content (0–30, 30–60, and 60–90 cm). In the h-mode the calculation of clay, the upper depth range was better reflected than at deeper levels with an R^2^ of 0.38. The coefficients of determination of the v-mode increased with increasing depth from 0.13 to 0.22. In both cases, the enhancement was only slightly pronounced. The relationships were still weaker when calculating soil water content. 

### 3.4. Measurements under Different Wetness Conditions

There is an ongoing discussion if there are particularly beneficial wetness conditions for the measurements. According to [11], it is not possible to give a single answer. On fields with distinct soil texture differences, the margin of the EC_a_ distribution increases and is flatter during summer compared to winter readings. In fields with more homogenous EC_a_ distributions, the range became marginal closer during summer. The EC_a_ readings during wetter periods (>10 mm antecedent precipitation during the previous 7 days) showed greater spatial variability (shorter spatial correlation ranges and greater sills), indicating soil water influences the distribution of soil EC_a_ [77]. In wetter areas, a significant correlation between the relative difference of EC_a_ and that in measured soil moisture was observed (R^2^ = 0.59–0.77), but not in drier areas. During drier periods or at drier locations, the influence of soil moisture (and flow path) on EC_a_ was masked by the terrain and other soil properties. This means that the optimal use of EM38 for detecting subsurface water contents would be during wet periods or in wet areas. The authors recommend mapping soil C, and the influence of soil moisture on EC_a_ should be minimized. A survey conducted during a dry period may yield a better result. 

According to our experience (unpublished) from a hilly site of Tertiary and Quaternary material, a generalization is not possible. At our observation area, the EC_a_ values decreased from April to July (April 29 mSm^−1^, July 19 mSm^−1^). The strongest decrease indicated EC_a_ readings in the subarea ‘Tertiary clay covered with a layer of 40 cm of sand-gravel’ (fine Vertic Eutrochrept). No reduction or only small decreases occurred in areas with Pleistocene loesses (fine-silty Dystric Eutrochrept, fine-loamy Typic Udifluent). Other sites showed more negligible reductions (e.g., coarse-silty Dystric Eutrochrept, fine-loamy Dystric Eutrochrept). 

Other authors (Maier et al. 2006) [78] investigated if a variation of the soil conductivity resulting from changes in the soil moisture influences the measured susceptibility values. A laboratory experiment indicated a weak variation in the measured magnetic susceptibility under different water contents. A measurement error, caused by an interfering effect of soil conductivity variations, was not found. The authors concluded that in practical applications for topsoil magnetic susceptibility mapping in the field, the influence of soil moisture is less important. 

### 3.5. Modelling EC_a_ Gradient (with Increasing Depth) from EM38 Readings 

Detecting vertical variations of soil properties are essential for soil dynamics, rooting depths and water turnovers. One possibility to assess the vertical discontinuity is the measurement in the vertical and horizontal mode. However, only few researchers have attempted to derive more detailed vertical changes with ECa readings. In geophysics, the procedure to survey soil with a larger number of penetration depths is called the vertical electrical sounding [79]. 

One of the first vertical electrical sounding was performed with resistivity sensors, where the investigation depth is controlled by the spacing of the electrodes [80]. For EM38 with a fixed coil distance, the only possibility to detect layering is the coil orientation, measurements at different heights above the ground as well as the combination with other sensors. With increasing heights, the response of air increases, and the influence of the soil decreases [39,79]. Mester et al. (2011) [81] combined the measurements of EM38 and the Profiler system with 1.22 m offset and 8 and 15 kHz for calculating lateral and vertical conductivity variations. 

Frequently applied here is the inversion-calculation-technique. Inversion of EC readings need solving the matrix formula for the not known EC profile (σ) [12,36,82]:

(35) Kσ=d

K: matrix of the depth response functions of the instrument at different heights
d: vector of ECa values.

In the eighties and nineties, several authors described empirical relations between aboveground EC_a_ and conductivity soil data (e.g., [83,84,85,86]). McNeill (1980) [9] published two depth response functions for EM38. [82] used the same model with a second order Tikhonov regularization. All of these relationships were based on the assumption of linearity [36]. Cook and Walker (1992) [15] developed an optimal linear combination depth weighted function to determine EC_a_ for a specific depth using a least square minimization. [87] attempted to reconstruct horizons and layers with EC_a_ measurements. 

Verwoort and Annen (2006) [88] tested three different inversion methods described by [9], the Tikhonov regularization discussed in [82] and the method developed by [15]. The calculations produced small differences in K_s_-values stratigraphies. After conversion, the last method and Tikhonov method appeared to represent the area wide Ks distribution more accurately than the first procedure.

The most actual form of the calculation is inverse modelling combined with the nonlinear procedure and Tikhonov regularization [36,88]. Some authors used older forms of the calculation, e.g., linear modelling [89]. Gebbers et al. (2007) [79] applied different inversion programs/algorithms (IX2D (Interpex), lsqnonneg (MATLAB solver), fminunc (MATLAB solver), and GPCG).

Hendrickx et al. (2002) [36], using data from agricultural fields in California, obtained adequate results using linear and nonlinear inverse procedures. Using linear inverse modelling, [89] described a good conformity in the topsoil of a rice-paddy soil in the Yangtze Delta, China with validation data. [79] found that the inversion was uncertain because of the principle of equivalence and measurement errors. The authors did not observe large differences between inversion algorithms. The GPCG procedure only calculated better results without regularization with error-free data.

Triantafilis and Monteiro Santos (2009) [90] inverted EM38 data in a two-dimensional (2-D) image of the true conductivity distribution. The authors found favorable relationships for the cation exchange capacity, particle size fractions and the inverted EM38 values. However, some of these comparisons were not satisfactory. The difference between these results was that the former study site was located on soil derived mainly from sandstone, which delivered conductivity values below 100 mSm^−1^, whereas the latter was located on clay plains where the conductivity data were commonly 100 mSm^−1^ or greater. 

Li et al. (2010) [89], (2013) [91] developed a combination of the Tikhonov regularization and a 3-D anisotropic variogram. The results allowed the prediction of a three dimensional spatial variability of EC_a_.

Section 3.1.3 shows that the magnetic susceptibility readings (i/p-phase, inphase) are used, especially in archaeology. In addition to area wide sensing, Dalan and Bevan (2002) [61] performed a series of measurements as the EM38 was dropped from a height of 2 m to the surface at intervals of 1 cm or 5 cm. Susceptibilities of the soil samples were measured using a Bartington MS2 susceptibility meter and MS2Blabsensor. 

In summary, until now it is not possible to recommend a practicable procedure (EM38 in combination with other tools). However, today a new generation of multi-configuration EC_a_ tools is commercially available. These systems have the similar construction (transmitter and receivers) but with different coil distances and coil orientations. Such systems enable the simultaneous sensing over different depth ranges 

### 3.6. Additional Aspects of Special Applications

This section includes different additional aspects for the application of EM38 and to process EC_a_ readings.

- ECa measurements do not provide absolute values of electrical conductivity because of calibration problems, which prevent a quantitative analysis of the readings [4,92,93]. This means that the combination of different maps over different times can be difficult to assemble because of shifts in the relative values. Calibration is a general problem not limited to EMI devices and some users have developed calculations to transform results in absolute physical units.
  Lavoué et al. (2010) [92] described the procedure to calibrate ECa induction measurements with electrical conductivity values measured with electrical resistivity tomography (ERT). The inverted ERT data were used as input in a forward modelling tool considering the frequencies and coil distances. Comparison of the calculated and measured apparent electrical conductivities showed very similar trends but a shift in absolute values.
  Moghadas et al. (2010) [94] described a conceptual EMI model for a zero-offset using vector network analyser technology. Theoretically the modelling approach is exact, but not yet applied to a real field. The correction routine of [35] included for measured ECa data by examining the theoretical relationship between the commercial ECa system and the level of subsurface conductivity, coil configurations and the instrument elevation.
  The approaches listed represent useful procedures to determine the electrical conductivity of the soil and are not very time-consuming compared to the duration of the normal-size EC_a_ survey. The methods turn the ECa readings from a proxy indicator toward a more valuable level ([95] that quantitatively characterizes the ground. However, the performance on highly resistive areas as well as on areas with less vertical differentiation needs further measurements [95].
- A moderate weakness was reported by [68]. These authors ascertained that each EM38 device has a slightly different response.
- When towing the instrument too fast (>1 m/s) a delay between the GPS registration and the measured ECa can occur.
- Measurements are highly affected by the height above the ground at which the EM38 was held. The effect of readings depends on the relative height of the tool, and the actual conductivity at each depth. Korsaeth (2006) [8] developed a correction function (EC_av-corr_, EC_ah-corr_) for measurements conducted at some height above the ground:
  (36) $${\mathrm{EC}}_{\mathrm{ah}-\mathrm{corr}}={\mathrm{EC}}_{\mathrm{ah}}\;\times \;\frac{1}{{\left(4\times \left(-h^{2}\right)+1\right)}^{0.5}\;}-2\times \left(-h\right)$$
  (37) $${\mathrm{EC}}_{\mathrm{av}-\mathrm{corr}}={\mathrm{EC}}_{\mathrm{av}}\;\times \;\frac{1}{{\left(4\times \left(-h^{2}\right)+1\right)}^{0.5}\;}$$
  h: height (m) above ground
  where the subscript corr indicates the height (h) corrected EC_a_-data.
  For simplicity, the author assumed that the soil profile has a uniform conductivity. At heights 20 cm above the soil surface, the corrections were sufficient. However, the methods have not been tested for heights below 20 cm.
- For most surveys the instruments are placed as close to the ground as possible. However, increasing the height of the instruments reduces the magnitude of the EC_a_ readings and therefore the conductivity differences. This makes it harder to discriminate between soil conditions with different EC_a_. Morris (2009) [96] recommended the creating of a map with EC_a_ ratios which divide conductivity values throughout a field by a typical conductivity in one area of the field. EC_a_ ratio maps make it easy to map spatial variations in conductivity.
- Lück et al. (2000) [11] described that if the position of the EM38 is not accurately vertical or horizontal, the readings show values between both modes. The authors concluded that on fields with small scale floor unevenness, measurements have severe fluctuations.
- In our experience (unpublished data), measurements closer than 2.5 m to a slope edge produce decreased readings. We assume that, close to the edge, the half-space of the soil below the device consists of air.
- Additionally, it is assumed that crop residues can influence EC_a_ values. Brevik et al. (2003) [97] compared EC_a_ readings collected above crop residues and bare soil. On average, EC_a_ readings were 0.2 mS m^−1^ higher when the EM38 was exposed to bare ground, and 68% of the bare ground readings were higher than the corresponding readings caused by crop residues. However, this difference was not significant when compared to the natural variation of the readings.
- The disadvantages of conductivity surveys include the EM38’s sensitivity to electrical interference (e.g., lighting, power lines) and metal debris [45]. In certain cases, however, the EM38’s sensitivity to metal is an advantage, such as at battlefields or other sites where metal artefacts are among the target features [98].
- Ernenwein et al. (2007) [99] described the application of EM38 to detect archaeological features. The authors assumed the following penetration depths:
- EM38, conductivity, vertical mode 1.5 m;
- EM38, conductivity, horizontal mode 0.75 m;
- EM38, magnetic susceptibility, vertical mode 0.5 m;
- EM38, magnetic susceptibility, horizontal mode 0.25 m;
- As a general rule of thumb, objects smaller than approximately 0.25–0.30 m are not detectable, except for magnetic materials. Other objects that are strongly magnetic (iron, nickel, magnetite, ferromagnetic material), even if very small, can sometimes be detected with EM38 if buried in the upper meter.

## 4. Comparison of EM38 with Other Conductivity/Resistivity Soil Sensors

There are several sensors for mapping soil conductivity commercially available in addition to the EM38, e.g., ARP03 (Geocarta, France), VERIS3100 (Veris Technologies, Salina, KS, USA), CM-138 (Gf Instruments, Czech Republic), MuCEP [100] (Panissod et al. 1998), soil doctor, DUALEM-devices (DUALEM Inc. Milton, Ontario) and GEM300. 

EM38, EM31, Dualem-2 and Dualem-4 meters and Veris 3100 were compared by [101] to assess the depth to sand and gravel. All five tools produced similar spatial patterns of EC_a_. The depths to sand and gravel were most strongly correlated with the measurements obtained with the EM31 meter in the vertical dipole orientation (R = 0.81), and the Dualem-4 meter with the horizontal coplanar geometry (R = 0.77). The authors concluded that variations in the degree of correlation between ECa and depth to sand and gravel demonstrate the importance of selecting an instrument and configuration that provide the maximum response for the depth of interest. 

Saey et al. (2008) [102] reported that the EM38 and Dualem-21S sensors had accurate depth predictions of a two-layered soil in a rapid, effective, and non-destructive manner. With the EM38DD, a number of soil auger observations are required to calibrate the EC_a_ measurements with respect to the depth of the layer. The quadruple-array DUALEM-21S instrument does not need to be calibrated with soil samples. 

Sudduth et al. (2010) [103] tested the EM38, VERIS3150 and DUALEM-2S sensors on Missouri claypan soils with five different EC_a_-depth-response functions to derive the topsoil depth. The results from the model-based approach were very similar to those obtained by regressing topsoil depth on EC_a_. The best calibrations were reached with multiple variables in the model inversion or regression compared to those with single variables. The authors did not prefer a single sensor.

Simpson et al. (2010) [56] compared the EM38 and the DUALEM-21S. First, the modelled sounding values agreed with the measured data, meaning that the theoretical models are usable for layered soils. Second, the magnetic structures of buried objects were recognizable as anomalies with high resolution. These anomalies were detected with 2 m horizontal coplanar and 1 m perpendicular coil configurations. The detection of clay content and sand blows with different devices were described by [104] (EM38, GEM300 sensor, and a Veris 3100). All three sensors produced similar EC_a_ spatial patterns corresponding to mapped clay content. Sudduth et al. (2003) [105] compared the EM38 and Veris3100. On a single field and measurement date, EM38 data and Veris deep (0–100 cm depth) data were the most correlated (R= 0.74 – 0.88). Differences between EC_a_ sensors were more evident on more layered Missouri soils. The regressions of EC_a_ with depth–weighted clay content and cation exchange capacity were generally high. Significant R^2^ values were present with organic C and silt on the Missouri fields. Significant correlations of EC_a_ with soil moisture, sand content, and paste soil electrical conductivity were observed for approximately 10% of the comparisons. Selecting an EC_a_ sensing system should be based on practical implementation issues and the intended use of the data. Gebbers and Lück (2005) [5] and Gebbers et al. (2009) [106] concluded that the VERIS3100 and ARP03 were more suitable to obtain information from shallow depths than the v-mode from the EM38. When EM38 is used, one should consider the horizontal mode to investigate more shallow soil depths. Additionally, the authors stated that ground penetrating radar and EM38 react differently to layering. The EC_a_ values are influenced by conductive layers, and the ground penetrating radar readings are determined using resistive vertical discontinuities. [107] described a correlation between the EC_a_ measured by EM38, GSSI Profiler EMP-400 and Geocarta ARP and clay content. The R^2^ was higher with the ARP system. However, the correlation with EC_a_ and clay was strong for all sensors, except for the EM38-vertical mode. 

Fulton et al. (2011) [108] emphasized that the strength of the correlation may be affected by several factors, including how conductive the soil texture is relative to other soil properties. The authors described results which showed that on non-saline soils, EC_a_ measured with both EM38 and VERIS correlated with the content of gravel, sand, silt, and clay, but the strength of relationship between conductivity and these physical soil properties varied from moderately strong to weak. The application of EM38 and ground penetrating radar [109] to derive water content yielded a more site-specific image. These two geophysical data devices correlated at the silt loam site. However, the result from the loamy sand and the sandy site were distinctly weaker. 

Lilienthal et al. (2005) [110] expanded the comparison between EM38 and VERIS3100 and noted that EM38 was very sensitive to metal objects. The raw data needed to be screened for outliers. Even the corrected data showed structures from metal facilities, suggesting the use of data was questionable. The authors observed that the measurements of resistivity showed spatial patterns from fertilizing plots. 

Mankin et al. (1997) [111] reported that the meter’s Model SCT-1 (Martek Instruments, Inc., Irvine, CA), the conductivity sensor of Veris Technologies and the EC_a_ sensors (EM31 and EM38) produced valuable data to evaluate saline seeps and creating field-scale soil salinity maps. 

The juxtaposition of EM38 and EM31 were performed in rice fields in NSW, Australia [112]. The relationship between both devices was very strong. The authors believe that both instruments were suitable in the vertical mode. EM38 is cheaper to purchase than EM31 but has to be towed on a sledge close to the soil surface. This would cause wear and high maintenance and would not be suitable for cultivated rice fields. 

## 5. EM38 Fusion with Other Sensors

The idea behind the combination of proximal soil sensors is that the accuracy of a single sensor is often not satisfactory enough. The reading of one sensor is affected by more than one soil property of interest [113,114,115]. The fusion of sensor data can overcome this weakness by extracting complementary information from multiple sensors or sources. To an increasing extent, the readings of EM38 are evaluated in combination mainly with VIS–NIR and a gamma-ray-spectrometer. 

Mahmood et al. (2012) [116] described that soil property models based on the fusion of data from the EM38 and the VIS-NIRS (ASD FieldSpec) spectrometer can significantly improve the accuracy of predictions of soil properties, such as clay, silt, sand, soil conductivity (1:1) and pH compared to a single evaluation. The accuracy of predictions of total organic carbon, total nitrogen and C/N-ratio was better, but only for some fields. The authors described that the best predications were calculated on clayey fields and the worst were from sandy fields. 

Buchanan and Triantafilis (2009) [117] addressed the calculation of the water table depth using stepwise multiple linear regressions with different ancillary data sets (EM34, EM38, gamma radiometric) and morphometric data. The greatest improvement resulted when all data sets were combined (37% increase in prediction accuracy over interpolation of single measurements). Piikki et al. (2011) [118] combined gamma-ray spectrometer readings and EM38 and additionally relief values, radiance and distance to the next drainage system data with the target to map the clay content in the upper soil. The following results were summarized from the study:

(i) Clay content mapping was improved using EC_a_ measurements from multiple measurements;
(ii) Predictions based on EC_a_ data were improved by adding radiance data, but the addition of drainage or elevation data had no noticeable effect;
(iii) Predictions from gamma-ray-spectrometers were accurate and were not improved by adding EC_a_ or any other independent data.

Piikki et al. (2013) [118] recommended using EC_a_ maps as a decision support to interpret the soil heterogeneity of field trials. The fusion of EM38 and gamma-ray-spectrometers is also described by [119]. The authors found an improvement when predicting the topsoil depth and pH. Using the same sensors, Taylor et al. [120] (2010) concluded that the combination of sensors predicted topsoil clay content better than a single application. Rodionov et al. (2013) [121] indicated no gradation between the devices. For similar conditions (e.g., water content), both sensor signals are proxies for soil texture and can be used complementarily. The use of both techniques would reduce the uncertainties of EM38 measurements. 

De Benedetto et al. (2013) [122] used EM38 and ground penetrating radar to calibrate a field wide map of soil water content. Both readings were processed using multi-collocated co-kriging. The results indicated that a multivariate geostatistical approach is effective to fuse sensor information to estimate soil water content. 

GEOPHILUS ELECTRICUS [123] is a novel system for mapping the electrical bulk resistivity of soils. The comparison with VERIS and EM38 indicated a similar pattern which can be associated with the remnants of a former river channel. All datasets show sharper contrasts for the whole depth range than for the contrasts associated with the upper horizon. GEOPHILUS data are well correlated with the data recorded using the EM38 and VERIS system. 

## 6. Closing Remarks and Future Research

Experimental explanations and theoretical and practical results have been presented and discussed to contribute to an improved application of the EM38. 

The knowledge of the most influencing external factors is essential in the daily practice and can avoid misunderstanding and misinterpreting conductivity values. For example, the EC_a_ distribution on a field cannot distinguish between sandy soils and gravels, which have similar and low EC_a_. 

The measurement of EC_a_ with this tool is complicated because of the influence of external and internal factors. Moving the in-phase and quad-phase values makes the application difficult for less-experienced users. Low values of EM38 deliver uncertain measurements. The temperature of the sensor and the environment represent influencing factors. EM38 requires a relatively frequent tuning, whereas the widely used resistivity instruments, after the initial setup, require no calibration. This paper addresses procedures to minimize these disadvantages. The manufacturer has now equipped the new instruments with automatic compensation (IP and QP), but until now, no results have been published. 

EM38 is useful for several purposes because the readings can be affected by many different soil parameters. However, the application of the EM38 requires site-specific calibrations.

Additionally, a possible drawback is that the investigated half-space is too large for the root zone.

The advantages and disadvantages of different sensors have been discussed but the applications and procedures were not ranked. Compared to current systems (resistance tools), EM38 measurements can be performed more quickly because it is not necessary to insert probes into the soil. This means that EC_a_ readings can be collected in areas with dry or hard soil surfaces, where currents will not flow because there is not enough moisture or it cannot be inserted in the soil. Compared to resistance sensors and alternating current instruments, EM38 is easier to handle because of its small size. The EM38 is very easy to handle (1.5 kg), and the VERIS and ARP systems need a towing vehicle.

The next logical step is to combine sensors to develop increasingly sophisticated applications. Sensor data fusion may provide many possible benefits, such as robust accuracy, extended attribute utility and complementary information on certain soil properties. There is a need for more optimal handling of statistical and geostatistical data evaluation. A literature review shows that there is scope for better soil property estimates using sensor data fusion. This approach has not been intensively tested for predicting multiple soil properties. The listed sensor combinations with gamma-ray or VIS-NIRS techniques are limited to the soil surface or a few cm of penetration depth. Sensor data fusion can enhance the quality of soil sensing in precision agriculture once a proper set of sensors has been selected for fusion.
